# Cost-Effective, Safe, and Personalized Cell Therapy for Critical Limb Ischemia in Type 2 Diabetes Mellitus

**DOI:** 10.3389/fimmu.2019.01151

**Published:** 2019-06-04

**Authors:** Bárbara Soria-Juan, Natalia Escacena, Vivian Capilla-González, Yolanda Aguilera, Lucía Llanos, Juan R. Tejedo, Francisco J. Bedoya, Verónica Juan, Antonio De la Cuesta, Rafael Ruiz-Salmerón, Enrique Andreu, Lukas Grochowicz, Felipe Prósper, Fermín Sánchez-Guijo, Francisco S. Lozano, Manuel Miralles, Lourdes Del Río-Solá, Gregorio Castellanos, José M. Moraleda, Robert Sackstein, Mariano García-Arranz, Damián García-Olmo, Franz Martín, Abdelkrim Hmadcha, Bernat Soria, Collaborative Working Group “Noma Project Team”

**Affiliations:** ^1^Fundación Jiménez Díaz Health Research Institute, Madrid, Spain; ^2^Department of Regeneration and Cell Therapy, Andalusian Center for Molecular Biology and Regenerative Medicine (CABIMER), University of Pablo de Olavide-University of Seville-CSIC, Seville, Spain; ^3^Spanish Biomedical Research Centre in Diabetes and Associated Metabolic Disorders (CIBERDEM), Madrid, Spain; ^4^Andalusian eHealth Library, Sevilla, Spain; ^5^Unidad de Isquemia Crónica de Miembros Inferiores, Hospital Victoria Eugenia de la Cruz Roja, Sevilla, Spain; ^6^Servicio de Cardiología, Hospital Universitario Virgen Macarena, Sevilla, Spain; ^7^Clínica Universidad de Navarra, Pamplona, Spain; ^8^IBSAL-Hospital Universitario Salamanca, Salamanca, Spain; ^9^Department of Surgery, University of Valencia, Valencia, Spain; ^10^Cirugía Vascular, Hospital Universitario de Valladolid, Valladolid, Spain; ^11^Servicio Hematología y Hemoterapia, Hospital Clínico Universitario Virgen de la Arrixaca, Murcia, Spain; ^12^Herbert Wertheim College of Medicine, Florida International University, Miami, FL, United States; ^13^ISABIAL and Institute of Bioengineering, University Miguel Hernández de Elche, Alicante, Spain

**Keywords:** cellular medicaments, cell-based therapy, clinical trials, diabetes, critical limb ischemia, cost-effective

## Abstract

Cell therapy is a progressively growing field that is rapidly moving from preclinical model development to clinical application. Outcomes obtained from clinical trials reveal the therapeutic potential of stem cell-based therapy to deal with unmet medical treatment needs for several disorders with no therapeutic options. Among adult stem cells, mesenchymal stem cells (MSCs) are the leading cell type used in advanced therapies for the treatment of autoimmune, inflammatory and vascular diseases. To date, the safety and feasibility of autologous MSC-based therapy has been established; however, their indiscriminate use has resulted in mixed outcomes in preclinical and clinical studies. While MSCs derived from diverse tissues share common properties depending on the type of clinical application, they markedly differ within clinical trials in terms of efficacy, resulting in many unanswered questions regarding the application of MSCs. Additionally, our experience in clinical trials related to critical limb ischemia pathology (CLI) shows that the therapeutic efficacy of these cells in different animal models has only been partially reproduced in humans through clinical trials. Therefore, it is crucial to develop new research to identify pitfalls, to optimize procedures and to clarify the repair mechanisms used by these cells, as well as to be able to offer a next generation of stem cell that can be routinely used in a cost-effective and safe manner in stem cell-based therapies targeting CLI.

## Introduction

**Figure 1:**
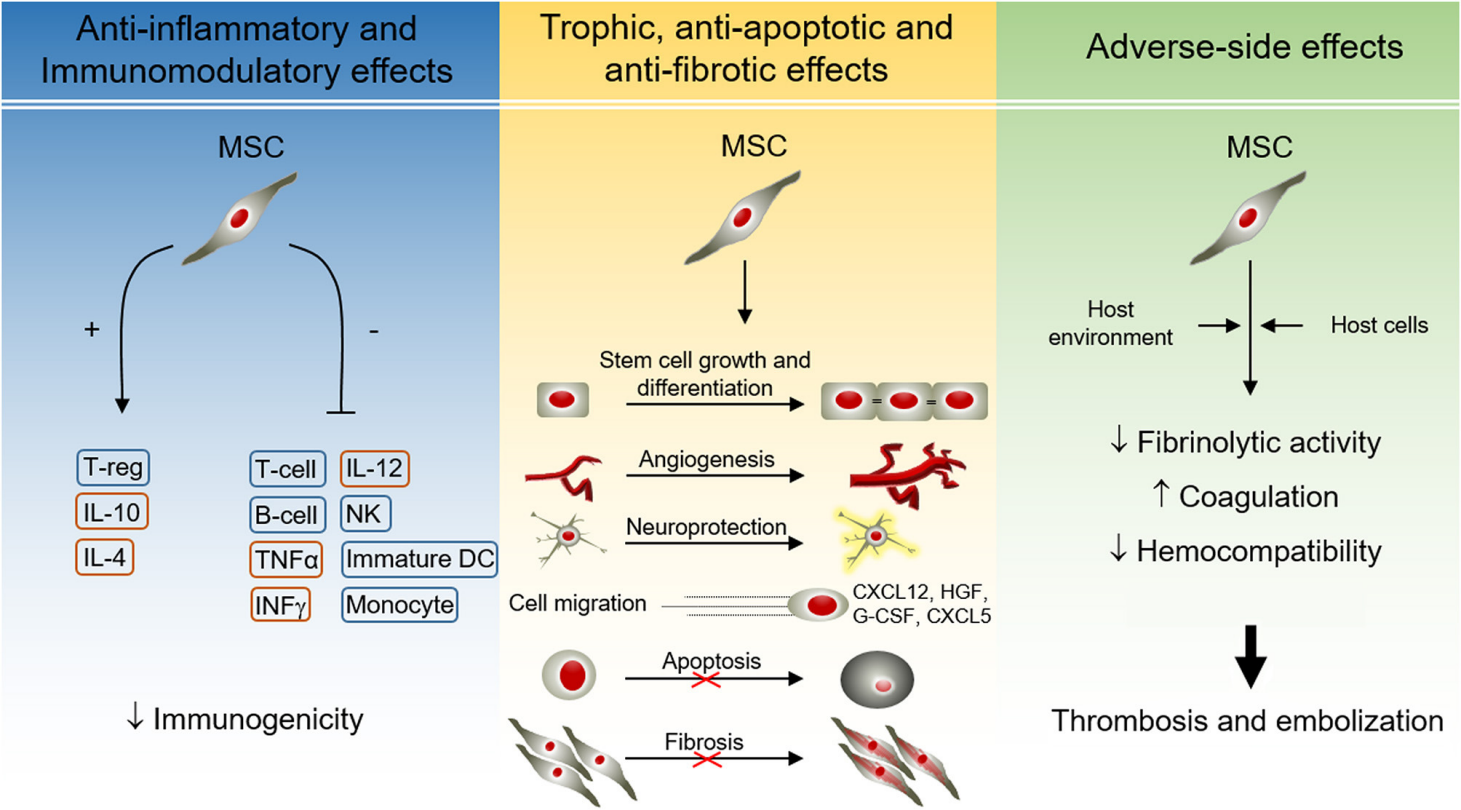
Effects of MSCs during therapeutic application. MSCs possess a broad range of paracrine effects, including anti-inflammatory, immunomodulatory, trophic, antiapoptotic, and anti-fibrotic properties. Most of them are mediated by molecules released by MSCs, but also by direct cell-cell contacts. The paracrine properties of MSCs have beneficial effects during cell therapy for regenerative medicine. However, the interaction between MSCs and the host may result in adverse-side effects, including thrombotic events. *B-cell*, B lymphocyte; *CXCL*, C-X-C Motif Chemokine Ligand; *DC*, dendritic cell; *G-CSF*, granulocyte colony-stimulating factor; *HGF*, hepatocyte growth factor; *IL*, interleukin; *INF*γ, interferon γ; *MSC*, mesenchymal stem cells; *NK*, natural killer cells; *T-cell*, T lymphocyte; *TGF*α, transforming growth factor α; *T-reg*, regulatory T cell.

Regenerative Medicine is a new paradigm that has driven the revisiting of our understanding of biological and medical processes and suggested new treatments. According to the definition of the European Medicament Agency (EMA) and the U.S. Food and Drug Administration (FDA), Advanced Therapies include Cell and Gene Therapy and Tissue Engineering. Advanced Therapies comprise a large group of translational fields and targets in areas of unmet medical needs. Briefly, the application of cells, either alone or engineered, as a pharmacologically active substance seeks to restore the functioning of damaged tissues or organs through the protection of cellular integrity, the replacement of damaged cells, and the promotion of trophic, anti-inflammatory, and immunomodulatory effects, among others (Figure 1). This new therapeutic avenue also carries unknown side effects that must be deeply characterized to improve safety, feasibility, and efficacy.

In this regard, mesenchymal stem cells (MSCs) are the cell type commonly used in Regenerative Medicine due to their unique biological properties, including ease of expansion and culture. The predominant sources of stem cells are summarized in Table 1, namely, cells derived from the fetus, and adult tissues (1). Nine hundred forty-one studies using MSCs have been reported to date (March 2019) and registered in the database of privately and publicly funded clinical studies conducted worldwide at “*ClinicalTrials.gov*.” MSCs are multipotent non-hematopoietic progenitor cells with different degrees of stemness, derived from the mesodermal germ layer and resident in most tissues (Table 1). This type of cell (MSCs) can be easily expanded *in vitro* because of their fibroblastic characteristics and ability to adhere to plastic and to express specific surface marker patterns (2, 3). The Mesenchymal and Tissue Stem Cell Committee of the International Society for Cellular Therapy (ISCT) first proposed that bone marrow plastic-adherent cells generally described as “*mesenchymal stem cells*” should be defined as “*multipotent mesenchymal stromal cells*,” while the designation “*mesenchymal stem cells*” should be reticent for a subset of these cells that show stem cell activity based on clearly stated criteria (3). Since the acronym MSC may be used to define both cell populations, the combined definition “*mesenchymal stem and/or stromal cells*” is probably more appropriate, especially when the “*stemness*” of the whole MSC population has not been demonstrated (4), and it is now widely accepted that MSCs represent a heterogeneous population (5) but are considered a cellular medicament. Furthermore, MSC survival, permanent engraftment and differentiation into resident cells were initially thought to be necessary to obtain the beneficial effects of these cells, and clinical experience and several experiments have shown that one of the primary functions of MSCs, most likely their key function, is to secrete several bioactive molecules related to the environmental “*niche*” in which these cells are located. Consequently, the secretome transiently reproduces most of the effects of MSCs, and in this sense, MSCs secrete a wide variety of pro-inflammatory and anti-inflammatory cytokines, chemokines, growth factors, and prostaglandins under resting and inflammatory conditions (1, 6) (Figure 1).

**Table 1:** Human stem cell sources and subtypes.

**Source**	**Tissue**	**Stem cells derived and acronyms**	**Brief definitions**
Fetal Newborn	Abortus (fetal tissues) Extra-embryonic tissues: Umbilical cord Wharton's jelly Amniotic membrane Amniotic fluid Placenta	Fetal stem cells (FSCs) Fetal structures like Wharton's jelly-derived mesenchymal stem cells (WJ-MSCs) Amniotic membrane-derived mesenchymal stem cells (Am-MSCs) Yolk sac-derived mesenchymal stem cells (YS-MSCs) Umbilical cord-derived mesenchymal stem cells (UC-MSCs) Umbilical cord blood-derived mesenchymal stem cells (UCB-MSCs) Amniotic fluid-derived mesenchymal stem cells (AF-MSCs)	Fetal stem cells are multipotent stem cells isolated from two distinct sources, the proper fetus (fetal tissues), and the supportive extra-embryonic tissues. These cells are also known as “primordial germ cells” and are isolated from tissues of 5- to 9-week fetuses obtained by therapeutic abortion. The three most reliable sources to date of abundant fetal stem cells are the placenta, amniotic fluid, and umbilical cord blood.
Adult	Bone marrow Peripheral blood	Hematopoietic stem cells (HSCs) Endothelial progenitor cells (EPCs) Bone marrow-derived mononuclear cells (BM-MNCs) Peripheral blood-derived mononuclear cells (PB-MNCs) Recombinant human granulocyte colony-stimulating factor (G-CSF)	Hematopoietic stem cells are the stem cells that give rise to other blood cells (hematopoiesis), a limited number of hematopoietic stem cells are multipotent and capable of extensive self-renewal. Endothelial progenitor cells define a group of cell population types with angiogenic activity. Endothelial progenitor cells can be obtained from the bone marrow-derived mononuclear cells fraction or from peripheral blood, and they can also be found in umbilical cord blood. Typically, endothelial progenitor cells are selected by isolation and enrichment strategies focused on the expression of surface markers CD34 and CD133.
	Bone marrow stroma Peripheral blood Adipose tissues: Fat, liposuction Others tissues: skin, gut, hair follicles, skeletal muscle, cartilage, tendon, synovium, perichondrium, cardiac tissue, oral cavity, dental pulp, salivary glands, etc.	Mesenchymal stem cells (MSCs) Bone marrow- derived mesenchymal stem cells (BM-MSCs) Peripheral blood-derived mesenchymal stem cells (PB-MSCs) Adipose tissue-derived mesenchymal stem cells (Ad-MSCs)	Mesenchymal stem cells are multipotent stem cells that can differentiate into a variety of cell types, including osteoblasts, chondrocytes, myocytes, and adipocytes.

*Summary of fetal and adult stem cells subtypes. Modified from Hmadcha et al. (1)*.

These molecules are associated with immunomodulation [indoleamine-2,3-dioxygenase (IDO), prostaglandin-E2 (PGE-2), transforming growth factor beta (TGF-β), human leukocyte antigen-G5 (HLA-G5), and hepatocyte growth factor (HGF)], anti-apoptosis [vascular endothelial growth factor (VEGF), granulocyte-macrophage colony-stimulating factor (GM-CSF), TGF-β, stanniocalcin-1 (STC1), and insulin-like growth factor 1 (IGF-1)], angiogenesis [VEGF, monocyte chemoattractant protein 1 (MCP-1), and IGF-1), local stem and progenitor cell growth and differentiation support (SCF complex, angiopoietin-1, and stromal cell-derived factor 1 (SDF-1)], anti-fibrosis [HGF and basic fibroblast growth factor (bFGF)] and chemoattraction [chemokine (C-C motif) ligand 2 and 4 (CCL2, CCL4), and C-X-C motif chemokine 12 (CXCL12 also called SDF1)] (7). Immunomodulatory properties of MSCs and their immunoprivileged condition make these cells good candidates for use in several clinical trials related to chronic, inflammatory and autoimmune diseases, and in reducing the incidence and severity of graft-vs.-host disease (GVHD). MSCs interact with cells of the innate or adaptive immune system (T cells, B cells, NK cells, monocyte-derived dendritic cells, and neutrophils). For a cell to be recognized by the immune system, the expression of major histocompatibility complex (MHC) and costimulatory molecules is necessary. MHC class I and class II human leukocyte antigens (HLAs) are master triggers of robust immunological rejection of grafts because they present antigens to cytolytic T lymphocytes (CTL). The interaction between MSCs and immune cells provides insights into *in vivo* MSC-mediated induction of tolerance (1, 8). MSCs display a low expression level of MHC-HLA class I, while they are constitutively negative for HLA-class II; likewise, they do not express costimulatory molecules such as CD80, CD86, CD40, and CD40L (9). However, MSCs share the expression of surface markers such as vascular cell adhesion protein 1 (VCAM-1), intercellular adhesion molecule 2 (ICAM-2), and lymphocyte function-associated antigen 3 (LFA-3 or CD58) with the thymic epithelium, which are crucial for the interaction with T cells (9, 10). Whereas, MSCs remain in a quiescent state showing antiapoptotic properties and contributing to homeostasis, in an inflammatory environment (presence of IFNγ, TNFα, IL-1α, and IL-1β) they begin to exercise their immunomodulatory abilities, inhibiting the proliferation of effector cells and their cytokine production. Similarly, MSCs can block a variety of immune cell functions (1, 11) (Figure 1).

In addition, there is a complex “*cross-talk*” interaction between MSCs and endothelial cells. MSCs increase the proliferation and migration of endothelial cells to promote the early events of angiogenesis and to decrease the permeability of the endothelial cell monolayer. In direct cocultures of MSCs and endothelial cells, MSCs increase the persistence of preexisting blood vessels in a dose-dependent manner (12). Additionally, beneficial therapeutic effects of the use of conditioned media of MSCs have been reported, which has even been shown to be therapeutically superior to the cells themselves (13, 14) and to stimulate the proliferation of local endothelial cells (15). Likewise, in addition to direct “*cell-cell*” contact, there has been speculation of a possible transfer of mitochondria or vesicular components (secretome) that contain mRNA, microRNA and proteins (16). Noteworthy, despite the anti-inflammatory, antiapoptotic, and immunomodulatory characteristics of MSCs, and due to their ability to migrate to sites of tissue injury and inflammation, many concerns have been raised about their probable precancerous activity (17). In this regard, the functions of MSCs can be influenced by the existing microenvironment, making them acquire supportive properties toward cancer cells (8, 18). To date, no cancer has been diagnosed or has recurred in clinical trials that would originate from experimentally given MSCs. However, potential risks, related to the growth support and enhancement of undetected or “resident” cancer cells, do exist, thus the potential of tumorigenesis should be further explored and monitored to detect the possibility of tumorigenicity related to MSCs and likewise, the administration of MSCs-based therapies must be thoroughly evaluated (8, 17, 18). However, although these properties are generally attributed to all MSCs derived from different tissues, preclinical and phase I/IIa safety, and feasibility data also suggest that MSCs represent a potential therapeutic option for the treatment of Critical Limb Ischemia (CLI). Conversely, as mentioned above, evidence from different studies has suggested that MSCs from diverse sources are not identical and do not always achieve the same efficacy levels and desired outcomes. Thus, MSC effects may be influenced by the constant crosstalk between the graft and the host, which could affect the MSC fate potential. For instance, autologous MSCs from patients with inflammatory diseases (e.g., diabetes) may carry phenotypic modifications, promoting undesirable effects on the host when they come into contact with host signals (19). Here, we will provide relevant information and alternatives to possibly improve the use of MSC-based therapy to benefit type 2 diabetic patients with CLI.

## Critical Limb Ischemia and Diabetes

The term CLI is used for all patients with chronic ischemic rest pain, ulcers, or gangrene in the limbs attributable to objectively proven peripheral artery disease (PAD). PAD is associated with several clinical conditions, e.g., diabetes, hypertension, cardiovascular disease, hyperlipidemia, obesity, and stroke (20). CLI is an advanced form of PAD, which is responsible for a high rate of amputations and is a major cause of morbidity and mortality worldwide. The incidence of CLI ranges from 500 to 1,000 new cases per million every year in Western Europe and North America (21, 22), and this number is expected to grow due to the aging population with a longer life expectancy and progressive increase in the incidence and prevalence of diabetes. It is estimated that more than 200 million people are living with PAD worldwide (21), which is a common complication in patients with type 2 diabetes (23). These two factors have driven the development of a more severe degree of PAD. CLI is not a specific disease *per se*; rather, it represents a syndrome that may develop from distinct pathophysiological processes (24). Although CLI is primarily a clinic-based diagnosis, it should be confirmed objectively and early in the disease process, e.g., through the ankle-brachial index (ABI), toe-brachial index (TBI), first toe pressure (FTP), toe systolic pressure, or transcutaneous partial pressure of oxygen (tc*p*O_2_). Further computed tomography (CT), digital subtraction angiography (DSA), Doppler echocardiography and magnetic resonance (MR) angiography are important non-invasive modalities for assessing the severity of CLI (20, 25).

PAD is a condition that is characterized by atherosclerotic occlusive disease of the lower extremities. While PAD is a major risk factor for lower extremity amputation, it is also accompanied by a high likelihood for symptomatic cardiovascular and cerebrovascular disease. Although much is known regarding PAD in the general population, the assessment and management of PAD in people with diabetes is less clear and poses some special issues. Presently, there are no established guidelines regarding the care of patients with both diabetes and PAD (26), although revascularization remains the most important therapeutic option and the main objective of CLI treatment, either by open surgery or endovascular modalities (27). Diabetic patients habitually suffer from long-segment vascular obstruction, mainly of the calf vessels (28) in type 2 diabetic patients, and the anatomic extension and distribution of atherosclerotic occlusive disease make these patients poor candidates for revascularization, resulting in continued disease progression, amputation, and death (28).

Thus, far, pharmacological treatment for these non-options patients has not been shown to be effective in the CLI course (29, 30). In fact, amputation is routinely recommended for these patients as the only option, despite its obvious dysfunctional involvement, along with the associated mortality and morbidity (27, 29). Therefore, there is a need for new effective therapeutic alternatives for a large number of patients with CLI (31).

In the diabetic patient, leg ischemia develops earlier and with greater intensity than in other diseases (vasculitis, Buerger disease). It is estimated that 15% of diabetic patients will develop CLI, and in most cases, it will lead to amputation (32). Of even greater importance, arterial lesions usually affect the more distal vessels (29). This localization of lesions makes revascularization difficult, either surgically or endovascularly. Furthermore, the clinical presentation of diabetic patients is also different, as it entails a greater component of tissue loss and gangrene, as well as fewer clinical manifestation of pain due to the frequently associated diabetic neuropathy. Diabetic patients in particular have fewer physiological mechanisms of angiogenesis and reendothelization, and thus the course of the disease is more severe and accelerated (26). The chronic hyperglycemia present in diabetic patients results in vascular remodeling altering neovascularization (30), with deficient and/or aberrant angiogenesis (33). This phenomenon is partly due to the associated oxidative stress, underlying endothelial dysfunction, and lack of regeneration of the vascular endothelium (26, 34). In fact, neovasculogenesis is disrupted in diabetes and metabolic syndrome due to hyperglycemia and increased hemoglobin A1c (HbA1c), neuropathy, hypercholesterolemia, oxidized low-density lipoproteins (Ox-LDL), reactive oxygen species (ROS) or increased fatty acids (35–38). In healthy people, homeostatic mechanisms, by which the vascular supply increases to match metabolic demand, are activated (39). However, these mechanisms are frequently disrupted in patients with CLI, and the physiological response is not able to deliver the necessary amount of blood flow and oxygen to the affected limb, causing the arterioles of these patients to dilate to the maximum and become insensitive to provasodilator stimuli (39). This phenomenon, referred to as vasomotor paralysis, is considered the result of chronic exposure to vasorelaxation factors in patients with vascular diseases, which may explain the failure of most vasodilator therapies to improve functional capacity in individuals with PAD. Likewise, the blood vessels of patients with CLI present a decrease in wall thickness, cross-sectional area, and “*wall-to-cell wall*” ratio, among others.

The use of different types of stem cells in the treatment of CLI is aimed at stimulating neovascularization in the area of severe ischemia. The procedure consists of the administration of cells in the ischemic tissues, either intravascularly or intramuscularly (Table 2), to form new vascular structures and/or segregate a number of angiogenic factors that regulate the process and favor the recruitment of new cells (25, 40–51). The formation of collateral blood vessels is promoted in an effort to improve blood flow in ischemic tissue, as well as alleviate the symptoms of the disease and, in most cases, prevent amputation of the affected limb in patients who do not respond to conventional treatments. The main objective of this process is principally regenerative, restorative and anti-inflammatory (20, 25, 31). Despite the high prevalence and incidence of CLI in diabetic patients, most studies have excluded patients with diabetes or with high HbA1c (44, 47, 49, 50) (Table 2).

**Table 2:** Published studies using cell-based therapy to treat CLI.

**Clinical trial ID (www.clinicaltrials.gov)**	**Phase**	**Cell type**	**Route of administration**	**Diabetic patients**	**References**
NCT00872326	I/II	BM-MNCs	Intraarterial	Included	(25)
NCT00371371	I/II	BM-MNCs	Intraarterial	There is no data available	(40)
NCT00282646	I/II	BM-MNCs	Intraarterial	Included buerger disease	(41)
NCT01480414	I/II	BM-MNCs	Intramuscular	There is no data available	(42)
NCT00221143	I/II	PB-CD34^+^	Intramuscular	There is no data available	(43)
NCT00883870	I/II	BM-MSCs	Intramuscular	Type 1 Diabetic patients were excluded	(44)
NCT01595776	I/II	Bone marrow derived- CD133^+^	Intramuscular	There is no data available	(45)
NCT01065337	II	TRC	Intraarterial and Intramuscular	Included	(46)
NCT00392509	I/II	ALDHbr Cells	Intramuscular	Patients with HbA1c >8% were excluded	(47)
NCT00523731	I	NMPB-ACPs	Intramuscular	There is no data available	(48)
NCT00468000	II	Ixmyocel-T	Intramuscular	Patients with HbA1c >10% were excluded	(49)
NCT00533104	I/II	BM-MNC/PB-MNC	Intramuscular	Patients with HbA1c >7.5% were Excluded	(50)
NCT00721006	II	MESENDO	Intramuscular	There is no data available	(51)

*ALDHbr Cells, autologous bone marrow-derived Aldehyde Dehydrogenase-bright cells; Ixmyelocel-T, BM-MNCs (CD90^+^and CD45^+^/CD14^+^); Mesendo, combination of bone marrow-derived MSCs and EPCs; NMPB-ACPs, non-mobilized peripheral blood angiogenic cell precursors; PB-CD34^+^, peripheral blood G-CSF-mobilized apheresis CD34^+^ cells; TRC, tissue repair cells (expanded bone marrow cells enriched in CD90^+^ cells)*.

## Clinical Trials for Pad, Ischemia and CLI

### The Route of Cell Administration (Intraarterial vs. Intramuscular)

Since the publication of the Therapeutic Angiogenesis using Cell Transplantation (TACT) study (52), more than 70 reported clinical trials have been reported in patients with CLI. Only 25% of those studies included diabetic patients (25, 53). It is known that several types of stem cells, derived from different sources, have the propensity for vascular development, and could potentially be useful in the management of CLI (Table 3). Some of these cells have been used in preclinical models as well as clinically to treat this condition. Among them, mononuclear cells (MNCs) have been the most widely used (1, 8, 20), and despite significant steps forward in defining their potential for therapeutic purposes, further progress has been mired by unresolved questions around their definition, and mechanism of action and because of their heterogeneity (4, 54, 55).

**Table 3:** Registered clinical trials using cell-based therapy to treat CLI.

**Clinical trial ID (www.clinicaltrials.gov)**	**Phase**	**Status**	**Cell type**	**Route of administration**	**Patient condition**
NCT00904501	III	Completed	BM-MNC	Intramuscular	Patients with HbA1c >7.5% were Excluded
NCT01408381	II	Completed	BM-MNCs	Intraarterial	Non-diabetic
NCT00987363	I/II	Completed	BM-MNCs	Intraarterial	Diabetic
NCT01867190	I/II	Completed	BM-MNCs	Intramuscular	There is no data available
NCT00595257	I/II	Completed	BM-MNCs	There is no data available	There is no data available
NCT00498069	–	Completed	BM-MNCs	There is no data available	There is no data available
NCT01245335	III	Completed	Bone marrow-derived cells	There is no data available	Patients with HbA1c >10% were excluded
NCT00616980	I/II	Completed	CD34^+^	Intramuscular	There is no data available
NCT01584986	II	Completed	PB-ACPs	Intramuscular	There is no data available
NCT01351610	I/II	Completed	BM-MSCs	Intravenous	Patients with HbA1c >9% were excluded
NCT01484574	II	Completed	BM-MSCs	Intramuscular	Excluded diabetic patients
NCT01824069	I/II	Completed	Ad-MSCs	Intramuscular	There is no data available
NCT01257776	I/II	Completed	Ad-MSCs	Intraarterial	Diabetic
NCT01663376	I/II	Completed	Ad-MSCs	Intramuscular	There is no data available
NCT00919958	I	Completed	PLX-PAD	Intramuscular	Patients with HbA1c >9% were excluded
NCT00951210	I	Completed	PLX-PAD	Intramuscular	There is no data available
NCT01483898	III	Completed	Ixmyocel-T	Intramuscular	Patients with HbA1c >10% were excluded
NCT00955669	I	Completed	BM-MNCs and BM-MSCs	Intramuscular	Type 2 diabetic patients
NCT00518401	I	Completed	Mesendo	Intramuscular	There is no data available
NCT00913900	I	Completed	CD133^+^	Intramuscular	There is no data available
NCT01019681	I	Completed	UCB-MNCs	Intramuscular	There is no data available
NCT02474121	–	Available	Bone marrow-derived cells	Intramuscular	Patients with HbA1c >10% were excluded
NCT01837264	I	Active, not recruiting	BM-MNCs	There is no data available	There is no data available
NCT00956332	I/II	Active, not recruiting	MultiGene Angio	Intraarterial	There is no data available
NCT01049919	–	Ongoing, but not recruiting	BM-MNCs	Intramuscular	Patients with HbA1c >10% were excluded
NCT01745744	I/II	Ongoing, but not recruiting	Ad-MSCs	Intraarterial	Non-diabetic
NCT01386216	I	Recruiting	BM-MNCs	Intramuscular	Patients with HbA1c >10% were excluded
NCT02099500	I/II	Recruiting	AD-MSCs liposuction	Intramuscular	Patients with HbA1c >10% were excluded
NCT02915796	I	Recruiting	G-CSF CD133^+^	Intramuscular	Diabetic
NCT02140931	II	Recruiting	PB-ACPs	Intramuscular	There is no data available
NCT01833585	III	Recruiting	PB-MNCs G-CSF	Intramuscular	There is no data available
NCT02551679	II	Recruiting	PB-ACPs	Intramuscular	There is no data available
NCT02089828	–	Recruiting	CD34^+^ and PB-MNCs	There is no data available	There is no data available
NCT02234778	–	Recruiting	Ad-SVF cells	Intramuscular	There is no data available
NCT02805023	I/II	Recruiting	BGC101	Intramuscular	There is no data available
NCT01456819	II	Recruiting	BM-MSCs and BM-MNCs	Intramuscular	There is no data available
NCT02454231	II/III	Recruiting	EPCs and BM-MNCs	Intramuscular	Patients with HbA1c >7.5% were excluded
NCT02864654	I/II	Enrolling with invitation	ADRC from lipoaspirate	Intramuscular	There is no data available
NCT02863926	I	Not yet recruiting	Bone marrow-derived cells	Intramuscular	There is no data available
NCT02538978	III	Not yet recruiting	BM-MNCs	There is no data available	There is no data available
NCT02501018	II	Not yet recruiting	CD34^+^	Intramuscular	There is no data available
NCT02477540	I	Not yet recruiting	BM-MSCs	Intramuscular	Type 1 diabetic patients are excluded
NCT01686139	I	Not yet recruiting	Allogeneic BM-MSCs	Intramuscular	Type 1 and 2 diabetic patients
NCT03042572	II/III	Not yet recruiting	Allogeneic BM-MSCs	Intramuscular	There is no data available
NCT03056742	II	Not yet recruiting	Allogeneic BM-MSCs	Intramuscular	Diabetic patients are excluded
NCT02993809	I	Not yet recruiting	BM-ECs and PRPE	Intramuscular	Patients with HbA1c >7% were excluded
NCT00488020	I	Unknown	BM-MNCs	Intramuscular	There is no data available
NCT00434616	II/III	Unknown	BM-MNCs	There is no data available	There is no data available
NCT01446055	I/II	Unknown	BM-MNCs	There is no data available	Patients with HbA1c >7% were excluded
NCT01903044	I/II	Unknown	BM-MNCs	Intramuscular	There is no data available
NCT00539266	II/III	Unknown	BM-MNCs	Intramuscular	Diabetic and non-diabetic
NCT00922389	I/II	Unknown	G-CSF and PB-MNCs	Intramuscular	YES (controlled)
NCT02336646	I	Unknown	Allogeneic BM-MSC	Intramuscular	Type 1 diabetic patients are excluded
NCT01216865	I/II	Unknown	UC-MSCs	Intramuscular	Type 2 diabetic patients
NCT01558908	I/II	Unknown	ERCs	Intramuscular	Patients with HbA1c >8.5% were excluded
NCT00145262	II	Unknown	BM-MSCs and BM-MNCs	There is no data available	Patients with HbA1c >6.5% were excluded
NCT02287974	I/II	Unknown	MNCs and CD133^+^ and Ad-MSCs	Intraarterial	There is no data available
NCT02145897	I/II	Unknown	SVF and Ad-MSCs	Intramuscular and Intravenous	Patients with HbA1c >7% were excluded

*ADRCs, adipose-derived regenerative cells; Ad-SVF, adipose-derived stromal vascular fraction Cells; BGC101, mixture of cells enriched for EPCs and hematopoietic stem cells; BM-ECs, bone marrow-derived endothelial cells; ERCs, endometrial regenerative cells; Ixmyelocel-T, BM-MNCs (CD90^+^and CD45^+^/CD14^+^); Mesendo, combination of bone marrow-derived MSCs and EPCs; MultiGeneAngio, endothelial and smooth muscle cells; PB-ACPs, autologous angiogenic cell precursors; PLX-PAD, placenta-derived MSCs; PRPE, platelets rich plasma; UC-MSCs, umbilical cord-derived MSCs*.

We conducted a pilot prospective single-center study (NCT00872326), phase I/IIa, that aimed to assess the safety and efficacy of intraarterial administration of autologous bone marrow-derived MNCs (BM-MNCs) in 20 diabetic patients with CLI (25). As described by Isner and Asahara (56) and to guarantee the homing of a great number of cells, BM-MNCs were administered intraarterially into the most affected leg “*target limb*” as close as possible to the ischemic area. One year after BM-MNC infusion, there was a remarkable improvement in the clinical status of most of the target limbs. In addition, the infusion of BM-MNCs induced an unexpected benefit of an improvement in the healing process, not only for ulcers but even for minor amputations. Furthermore, early clinical benefits of cell infusion consisted of patients having a widespread perception of less limb pain, an increase in pain-free walking, and warmness in the target limb. Unlike other studies, the cell dose was 10 times smaller than the dose used by other groups. Moreover, and surprisingly, six diabetic patients reduced their need to inject insulin, probably due to a decrease in peripheral insulin resistance (25).

Similarly, the PROVASA study (Intraarterial Progenitor Cell Transplantation of Bone Marrow Mononuclear Cells for Induction of Neovascularization in Patients with Peripheral Arterial Occlusive Disease Study), a multicenter, double-blind, phase II trial (NCT00282646) with an estimated enrollment of 40 patients with ischemic rest pain or non-healing ulcers randomly assigned (1:1) to receive BM-MNC treatment or placebo, demonstrated that in patients with CLI, intraarterial administration of BM-MNCs does not increase ABI but promotes ulcer healing and reduces rest pain. Furthermore, repeated administration of functional BM-MNCs was required for successful ulcer healing associated with improved limb salvage (41). In this context, several studies have demonstrated the feasibility of intraarterial delivery of BM-MNCs, their beneficial effect on improving endothelial function (25, 57) and overall improvements of ischemic pain and ulcer healing (25, 58). However, the quality of evidence for efficacy is limited, as most studies lacked a proper placebo or sham group because of the invasive bone marrow harvesting procedure required to obtain the cells (59–61).

Although intraarterial and intramuscular injection of autologous BM-MNCs have shown similar results, and combined intraarterial and intramuscular transplantation is clinically feasible (62), to date, an overwhelming majority of clinical studies targeting PAD have relied upon intramuscular cell delivery (63). Intramuscular administration is easier and less invasive, and it results in a transient placement of cells in the ischemic tissue, whereas intraarterial infusion is designed to directly inject cells into peri-ischemic areas, which are considered to have sufficient oxygen and nutrients to support cellular functions. Both delivery methods have obtained promising results in the improvement of angiogenesis (64). Klepanec et al. (65) compared the therapeutic effects of intramuscular and intraarterial delivery of BM-MNCs in a randomized manner. There were no differences among functional parameters in patients undergoing intramuscular vs. intraarterial cell supply. Preclinical (66) and clinical (67) data from our group substantiate these remarks; nevertheless, our humble experience indicates that the route of administration depends on the type and the dose of cell to be administered. Deciphering how stem cells manage the countless signals required for revascularization will improve CLI recovery, Qadura et al. (31) proposed a combination delivery of multiple cell types within supportive bioengineered matrices as a new therapeutic strategy to target CLI.

### Comparison of the Cell Type

Apart from the route of administration (intramuscular, intraarterial, or combined), the most ideal cell type must be identified, and a better understanding of the effective subpopulation of stem cells is necessary as stem cells are a heterogeneous population. Some clinical trials have directly compared different cell populations (67–69) or used a combination of angiogenic stem cells. Huang et al. (68) evaluated the transplantation of peripheral blood-derived MNCs (PB-MNCs) in the treatment of diabetic patients with CLI who had received a subcutaneous injection of recombinant human granulocyte colony-stimulating factor (G-CSF) to mobilize progenitor cells, which resulted in clinical improvements including reduced limb pain and ulcers, as well as no adverse effects specifically due to cell transplantation and no lower limb amputation in the transplanted patients. Tateishi-Yuyama et al. (52) investigated the feasibility and safety of intramuscular injection of MNCs and showed that in two groups of patients, the first with unilateral ischemia infused intramuscularly with BM-MNCs in the ischemic limb and saline in the less ischemic limb, and the second with bilateral leg ischemia receiving random intramuscular injections of BM-MNCs in one leg and PB-MNCs in the other as a control, resulted in significant improvements in patients treated with BM-MNCs compared with those treated with PB-MNCs in terms of ABI and rest pain. Lu et al. (69) compared the therapeutic effect of autologous intramuscular administration of bone marrow-derived MSCs (BM-MSCs) with BM-MNCs in 20 diabetic patients with CLI and foot ulcer. The authors demonstrated that the healing rate of ulcers was significantly higher in the group treated with BM-MSCs than with BM-MNCs. Likewise, the authors concluded that BM-MSC treatment was effective and better tolerated than BM-MNCs to improve lower limb perfusion and to promote foot ulcer healing in diabetic patients with CLI. Lasala et al. (51) evaluated the efficacy and safety of autologous intramuscular administration of a combination of MSCs and endothelial progenitor cells (EPCs) in 26 patients with bilateral CLI. They found that within this phase II clinical trial (NCT00721006), the enrolled patients experienced an increase in perfusion in the treated limbs compared with the control legs and improvement in pain-free walking time and ABI after cell infusion. In this context, our group proposed a phase I/II clinical trial (NCT02287974) to study and compare the therapeutic effect of autologous BM-MNCs, autologous BM-EPCs (CD34^+^/CD133^+^ cells) and autologous adipose tissue-derived MSCs (Ad-MSCs) on inflammatory and angiogenic cytokines, resistance to insulin and a decrease in the need for insulin, as well as evaluating the safety, viability and efficiency of the intraarterial infusion of these three stem cells types in patients with type 2 diabetes with CLI. We aimed to obtain related data on the source of suitable tissue, the most appropriate cell type, optimal dose of cells, efficient and low-cost protocols, among others, to be able to offer, in the near future, a high-quality, economic, and effective therapy for those patients without current therapeutic options. Despite the extraordinary and unpaid efforts of the clinical research team and promising preliminary results obtained during the first year of follow-up showing beneficial but distinct effects of cell type treatment, for unknown reasons the sponsor of this study decided to prematurely terminate patient recruitment, and we no longer have access to the clinical data.

### The Use of MSCs

Regarding the use of MSCs as a cell-based therapy for CLI, recent data suggest that the therapeutic effects of these cells in ischemic pathologies are due to the secretion of angiogenic molecules to bioactive levels and their ability to restore the microenvironment of the damaged area (70). In preclinical studies, the administration of autologous, allogeneic, and xenogeneic MSCs derived from various sources such as bone marrow, umbilical cord blood, fetal membrane and adipose tissue have been shown to be beneficial in rat and mouse models with lower limb ischemia (66). Subsequently, several phase I/IIa clinical trials have been assayed in a limited number of patients to demonstrate the safety and feasibility of MSCs obtained from different sources (71–75). MSCs isolated from healthy donors have shown uniform and consistent properties, whereas those from patients affected by degenerative and/or inflammatory disease differ in their biological and functional characteristics (8, 19, 76). In this regard, other studies using MSCs isolated from diabetic patients suggest that the hyperglycemic environment as well as other metabolic disorders associated with diabetes affect the cellular endogenous reserve and alter their proliferation, differentiation and angiogenic capacity (19, 77–79). Likewise, several groups have reported benefits of using autologous MSCs as a cell-based therapy for a wide variety of diseases, such as cardiovascular diseases (14, 31, 44, 51, 59, 61, 67, 71–75, 80–82), diabetic nephropathy (83), and diverse brain injuries including stroke, neural trauma, and heatstroke (84, 85).

Due to the combinatorial potential for inducing angiogenesis and the immunomodulatory effects *in situ* of BM-MSCs, Gupta et al. (44) reported the results of a randomized double-blind randomized placebo controlled multicenter phase I/II study examining the efficacy and safety of intramuscular administration of allogeneic BM-MSCs in 20 patients with CLI (NCT00883870). The administration procedure of BM-MSCs at a dose of 2 × 10^6^ cells/kg or placebo (PlasmaLyte A) was found to be feasible and safe; however, few of the efficacy parameters (such as ABI) showed significant improvements in BM-MSC arm transplant patients. Although immunogenicity may be unpredictable in cases where the administration of cells is used for a different function in the recipient than in the donor “*heterologous use*” or when injected into non-physiological sites, no evidence of harm or adverse events was detected with allogeneic administration of MSCs obtained from different source and prepared in different ways (81). This approach could result in a safe, feasible option to avoid the time involved in the process of isolation, expansion, and production of the use of autologous cells. Regardless, it is necessary to continue investigating this and other associated aspects to determine which cell type is best and feasible for a specific pathology and which type of patient health profile can benefit from this kind of cell-based therapy. Thus, improvements in cell therapy will benefit from a more precise characterization of cellular subsets in the therapeutic product.

## The Impaired Properties of MSCs Compromise Their Efficacy

Cell therapy is especially complex due to the nature of the product. The cellular source, isolation and expansion procedures, dose, site and procedure of administration define the cellular medicament, even without a precise knowledge of the mechanisms of action.

### Immunogenicity

The administration of stem cells could interact with the host immune response (for example, in a proinflammatory environment) or have an immunomodulatory effect. Although MSCs have been considered immune-privileged in this regard, long-term exposure to the culture medium can make them more immunogenic (MSCs are isolated and expanded in medium that contains fetal bovine serum (FBS) and/or human platelet concentrate), for example, by positively regulating the normal set of histocompatibility molecules (82, 86). Thus, allogeneic use of the cells may entail a greater risk of rejection by the immune system. This rejection could lead to a loss of function of the administered cells, and consequently, their therapeutic activity could be compromised. Nevertheless, use of immunosuppressants could limit these risks, but in turn could cause adverse effects due to immunosuppressive medication.

### Coagulation and Fibrinolytic Activity

MSCs have shown significant therapeutic potential due to their fibrinolytic and antithrombogenic properties (87–91). To date, several clinical trials have been conducted using autologous MSCs for the treatment of diabetes and its complications, which are presumably safer and more effective than allogenic cells. However, the therapeutic effects of MSCs have been questioned when they are derived from a diabetic milieu (19, 92–94).

**Figure 2:**
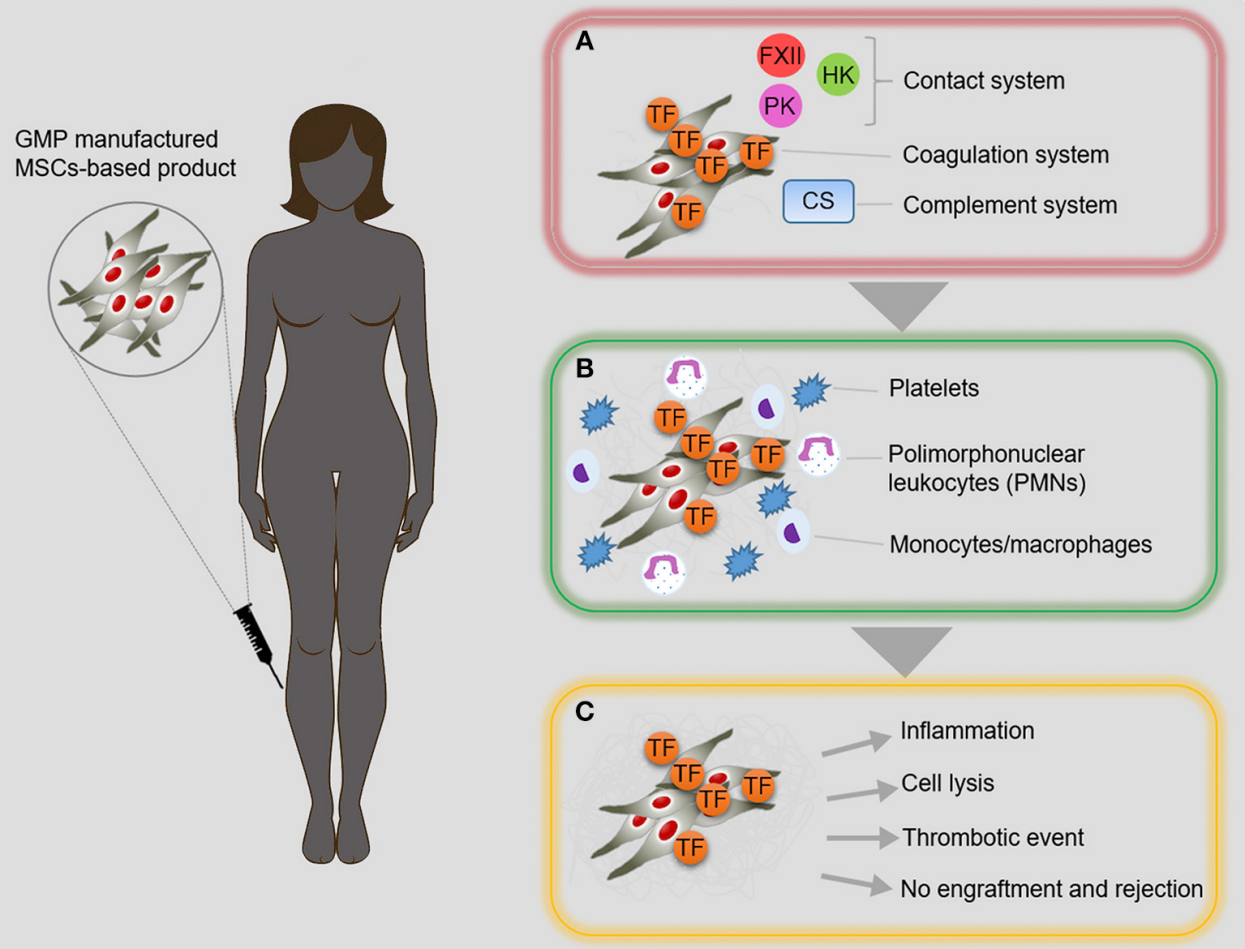
Instant blood-mediated inflammatory reaction. **(A)** The direct contact between intravascularly infused MSC-based products and blood stream, promotes inflammatory reaction known as instant blood-mediated inflammatory reaction (IBMIR). Adipose tissue-derived MSCs (AdMSCs) express tissue factor (TF/CD142) that mediates the activation of blood coagulation cascade and complement system, leading to inflammation and thrombotic reaction. **(B)** Consequently, multiple amplification reactions result in the activation of platelets and effector cells of immune system. **(C)** IBMIR is a multifaceted phenomenon that can compromise the success of MSC-based cell therapy. *CS*, complement system; *FXII*, factor XII; *HK*, high molecular weight kininogen; *PK*, prekallikrein; *TF*, tissue factor.

MSCs isolated from healthy donors have shown uniform and consistent properties, while those from patients, such as diabetic patients, differ in their biological and functional characteristics and can reduce the beneficial therapeutic effects of autologous MSCs (19, 76, 79). In this regard, studies carried out by our group and others using Ad-MSCs of diabetic patients suggest that the hyperglycemic environment and other metabolic disorders associated with diabetes affect the cellular endogenous reserve and their proliferation, differentiation, and angiogenic capacity, among other cellular characteristics (78, 79, 95, 96). Once infused in the recipient, the cells come into direct contact with the tissues, bloodstream and other patient cells, and the cell-recipient interaction process still requires thorough investigation and characterization. Physiologically, MSCs reside in the perivascular compartment of almost every tissue (97, 98); however, one of the hurdles to the sustained therapeutic success of these cells is early cell loss, which is largely thought to be due to incompatibility responses after systemic infusion of the cells, a reaction termed the instant blood-mediated inflammatory reaction (IBMIR) (63, 82, 86, 96–99). This reaction suggests that the immune and inflammatory system react to cells that normally are not in contact with the blood circulation (Figure 2). Moreover, it has been further shown that different MSC products display varying levels of highly procoagulant tissue factor, a decrease in tissue plasminogen activator (tPA) or an increase in plasminogen activator inhibitor type 1 (PAI-1) and may adversely trigger IBMIR or microthrombosis in the target tissue (19, 96).

MSCs are considered to be safe and even to promote fibrinolysis (88, 90). Since type 2 diabetes is a systemic inflammatory disease with a prothrombotic state, intraarterial infusion of Ad-MSCs in diabetic patients was suggested and approved by the Ethical Committee and the Regulatory Agency as the appropriate treatment within a clinical trial conducted by our group (NCT01257776), but unexpectedly during the course of this clinical study, two patients developed distal microthrombosis after intraarterial Ad-MSC infusion (19). These two patients reported oppression at the infrapopliteal level and vasomotor reaction of neurogenic origin in the distal third of the target limb, accompanied by discreet pain, 10 h after the cellular infusion. MR angiography demonstrated indemnity of the arterial vessels of medium caliber. These two patients were treated with antithrombotic therapy and discharged 72 h after the symptoms disappeared (19).

**Figure 3:**
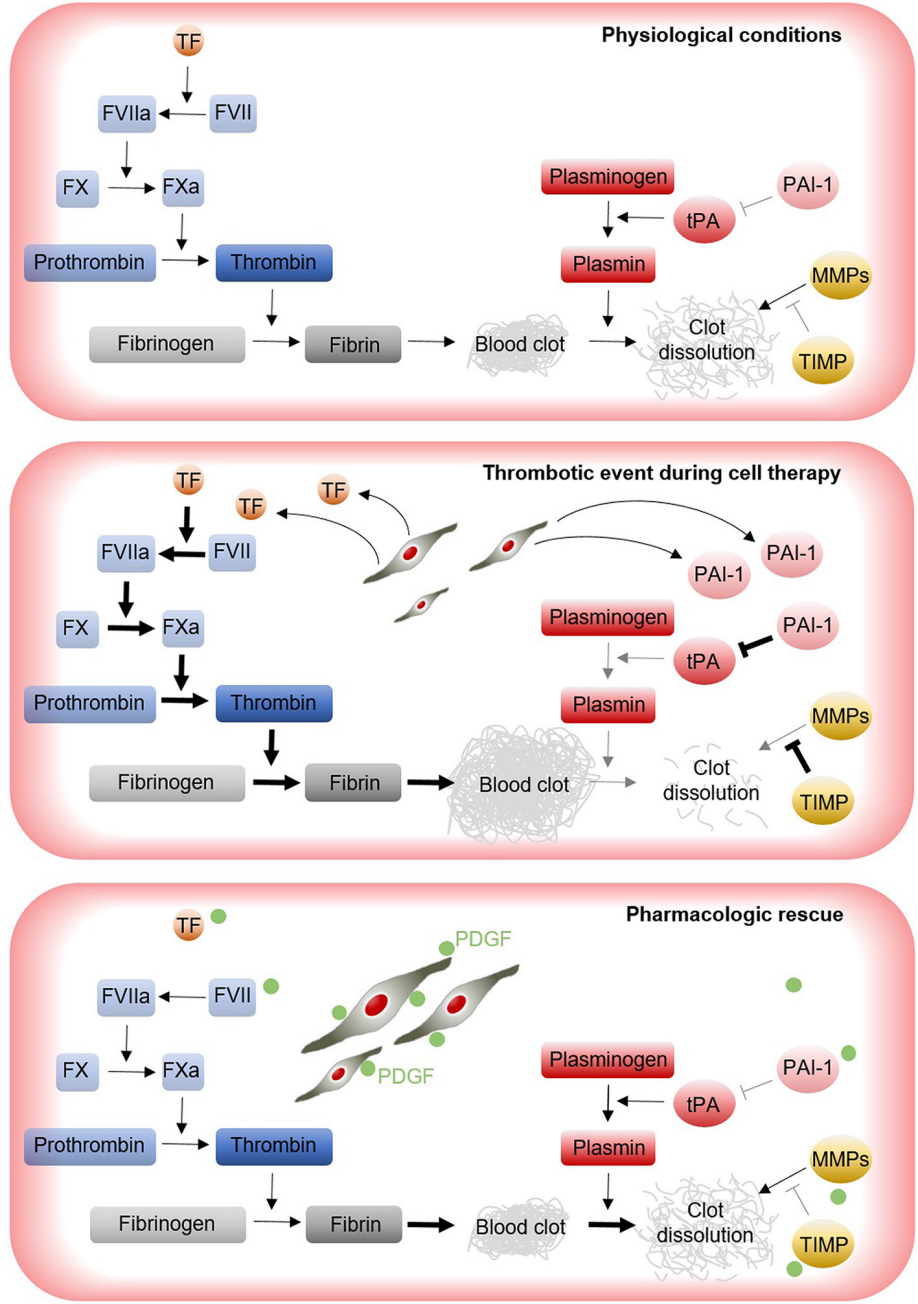
Implications of MSCs for thrombosis risk during cell therapy. Under physiological conditions, a delicate balance between the coagulation, and fibrinolysis cascades are responsible for the effective dissolution of the clots within blood vessels. During cell therapy, transplanted MSCs may induce the expression of pro-coagulant factors (e.g., TF and PAI-1) that disrupt the coagulation/fibrinolysis balance, increasing the formation of clots and leading to thrombotic events (19). Approaches aimed to produce more safety MSC products are being investigated. For instance, the pretreatment of MSCs with PDGF results in cell products with increased fibrinolytic activity, which may help to minimize thrombotic events during cell infusion (76). *FVII*, factor VII; *FVIIa*, factor VIIa; *FX*, factor X; *FXa*, factor Xa; *MMPs*, metalloproteinases; *PAI-1*, plasminogen activator inhibitor type 1; *PDGF*, platelet derived growth factor; *TIMP*, tissue inhibitor of metalloproteinase; *TF*, tissue factor; *tPA*, tissue plasminogen activator.

Furthermore, Ad-MSCs induced an increase in expression and release of PAI-1 and reduced levels of tPA. Likewise, the quantification of D-dimer also decreased. These responses were tested with MSCs of different origins exposed to different *ex vivo* environment. Effects were more pronounced when Ad-MSCs were from type 2 diabetic patients exposed to the sera of same patients (Figure 3). Therefore, the efficacy of fibrinolysis decreased favoring thrombosis, and these observations were published (19) and a patent filed to identify these tentative responses for cellular medicaments (US20160161504).

Altogether, new lines of research are being promoted that focus on enhancing the therapeutic effects of stem cells by regulating their biological characteristics (76, 95, 100–104). A recent study conducted by our group has demonstrated the beneficial effects of platelet-derived growth factor-BB (PDGF-BB) in restoring the defective phenotype of Ad-MSCs derived from type 2 diabetic patients with CLI (76). In particular, this study showed that pretreatment with PDGF-BB could potentiate proliferation, migration, and homing of defective MSCs, as well as recover their impaired fibrinolytic ability (Figure 3). Furthermore, we demonstrated that PDGF-BB utilized the ERK-SMAD pathway to exert its beneficial effects. Therefore, pretreatment with PDGF-BB represents a suitable strategy to produce more effective MSCs for autologous therapies (76). In this context, we postulated that either the use of allogenic MSCs from healthy donors, rescue of the healthy phenotype by pretreatment of autologous MSCs with PDGF (76), expression of Akt (103) or angiotensin 1 (104), or the use of early-passage autologous MSCs (105) will be an option to improve the therapeutic effect of the cells. While we have clinical data for the first scenario, the others have only been assessed in preclinical studies; since a side effect is coagulation, pretreatment with heparin (102) may overcome this problem.

## Considerations for an Advanced Therapy Medicinal Product

Safety, feasibility, and efficacy are mandatory to determine the viability of a clinical application for the treatment of any disease. With the exception of hematopoietic stem cell transplants, stem cell therapies used for the treatment of any disease are considered Advanced Therapies Medicinal Products (ATMP); therefore, their development, approval and use must be in accordance with specific standards established nationally and internationally for such products. Thus, regulatory authorities warrant the safety of the studies (8, 106).

Cell expansion and culture protocol are not yet standardized (4). Currently, there is no protocol or universal definition for stem cell culture and expansion Regulatory Agencies. For example, the Spanish Agency of Medicines and Medical Devices (AEMPS), the EMA and the FDA recommend a set of standards to be followed for the production of an ATMP (EMA: Regulation (EC) No 1394/2007 provides the overall framework on ATMPs and FDA: designation described in Section 3033 of the twenty-first Century Cures Act; cell therapy medicinal products are regulated in the Code of Federal Regulations under Title 21 PART 1271, Human Cells, Tissues and Cellular, and Tissue-Based Products). The different sources of origin, as well as the different methodologies for obtaining tissue cells, make it very difficult to compare research groups in search of the fastest, most effective, economical, high-yielding, and quality-required method.

Preclinical studies have shown that cell viability after infusion is quite limited and that very few cells survive after infusion. Although the *in vivo* follow-up in humans is ethically and technically complicated, it is necessary to continue investigating in this line to determine the intrinsic mechanisms of the integration of the infused cells in the specific microenvironment. Furthermore, the ATMP dose to obtain the desired effects remains to be determined and defined. Our previous clinical data (NCT01257776, NCT01745744, and NCT02287974) show that an intraarterial dose of 1 × 10^6^ cells/kg of body weight is more effective that a dose of 0.5 × 10^6^ cells/kg of body weight (107). Experiments using mouse as animal models have established a minimum dose of 1 × 10^6^ cells/kg of body weight, a quantity necessary to obtain a quantifiable but weak benefit. The dose for ATMP treatment is determined by the patient's body weight and the biodistribution of cells and paracrine factors secreted by the ATMP (MSCs) in the human recipient, and most clinical trials usually use a similar ATMP dose (108, 109). In most cases, the doses used for several clinical trials are likely not sufficient to achieve the desired outcomes and a clear therapeutic benefit. Therefore, collecting information regarding ATMP doses obtained from different sources and the influence of the host (patient recipient) medical conditions are important for proposing future clinical trials and will undoubtedly assure the safety and efficacy of ATMP-based therapies. Likewise, the frequency of administration is currently not yet determined, and the efficacy of ATMP-based therapy may be related to a precise number of repeated applications (40, 42, 110), as determined for a conventional medicinal product. Similarly, and in addition to the doses, the duration of ATMP application remains to be determined. Thus, ATMP-based therapy should be as close as possible to conventional medicines and thus may need to be adjusted accordingly. Finally, the method and route of administration of ATMP remains inconclusive, representing another variable to be considered in future clinical trials (96). From our perspective, the most suitable ATMP (defined by the cell type, culture media and standards, doses and route of administration) for a particular disease or complication remains a challenge for Regenerative Medicine and requires further investigation.

Therefore, the desired therapeutic effect depends on many factors since the mechanism of action of an ATMP in tissue regeneration is likely to be multifaceted; ATMP potency can be determined by the ability of the injected cells to migrate, survive, integrate, differentiate, and produce functional paracrine mediator factor involved in “*cell-cell interactions*.” As mentioned above, many diseases, including diabetes, affect the phenotypic and therapeutic properties of an ATMP, and in the search for safety and efficacy, the recipient tissue must respond favorably to the administered ATMP, which would result in the activation of endogenous regeneration mechanisms (111–114). Understanding the integration of exogenous mechanisms (injected ATMP) with the endogenous recipient (host) will play a decisive role in the future clinical use of adult stem cells (8, 96).

## New Generations of ATMP

Advances in compliance under good manufacturing practice (GMP) standards of more sophisticated cellular products are now paving the way for new ATMP generations for use in clinical trials.

The lack of therapeutic efficacy of the generation (Generation 1) of unmodified, naïve, and wild type MSCs rated in clinical trials can be explained by the observation that, after their systemic infusion (intravenous), these cells become trapped in vascular filters (fundamentally liver and lung), with only a small percentage reaching the target tissues. Therefore, it is essential that we design strategies that favor their migration, nesting, and localization in the inflammatory and/or infectious focus to increase their effectiveness (Generation 2 MSCs) (111, 113).

Biodistribution and long-term follow-up of these cells in animal models have shown that only a few cells persist after long periods of transplantation. This phenomenon supports the idea that most of the effects of MSCs are probably based on a “*hit and run effect*.” To increase the implantation of an ATMP in the injured tissue, Sackstein et al. (113), developed a method to transiently modify the CD44 antigen present in the MSC cell membrane by enzymatic fucosylation, converting this molecule into HCELL glycoform and thus favoring the migration of MSCs to the inflamed tissues (111, 115, 116). This method, called glycosyltransferase-programmed stereosubstitution (GPS) to custom modify cell surface glycans without affecting cell viability, has been optimized for its clinical application using an alpha-1,3-fucosyltransferase preparation and enzymatic conditions specifically designed to treat live cells and formulated to preserve the cell viability and phenotype. It has been found that this modification not only increases the adhesion of the MSCs to the endothelium but also enhances their transmigration through it by activating integrin α4β1 (VLA-4) in the absence of chemokine stimulation. Therefore, this modification by fucosylation could improve the efficacy of the treatment with MSCs by increasing their migratory capacity to the inflamed tissues after systemic administration. More detailed knowledge of the mechanisms of biodistribution, migration and specific interaction of MSCs at the damaged loci may be beneficial to design new ATMP with increased safety and efficacy (111), and in this regard, our group has collaborated in a patent to classify these tentative responses for stem cells (WO2017032612).

In this context, and as another approach, a new generation of MSCs can be engineered by increasing both cell migration and cell potency (Generation 3 MSCs). Targeting the CXCL12 and C-X-C chemokine receptor type 4 (CXCR-4) may improve the cell migration capacity of transplanted MSCs, and CXCL12 is also highly expressed in injured tissues and contributes to the recruitment of CXR4-positive cells. As a small proportion of MSCs express CXCR4 in culture, their capacity to migrate, and to respond to homing signals in damaged tissue may be reduced. Therefore, targeting CXCR4 may improve the migratory and therapeutic effects of MSCs (117). Within this context, we propose using MSCs modified to overexpress CXR4 and IL10 and/or IL7 (Generation 3 MSCs). Expression of the CXCR4 receptor will increase the migration of MSCs toward the inflammatory focus, while coexpression of the anti-inflammatory cytokine interleukin 10 (IL-10) and/or the anti-infectious cytokine interleukin 7 (IL-7) will increase the anti-inflammatory effect (IL-10) and even the anti-infective effect (IL-7).

Furthermore, we propose that the extensive use of FBS in MSC expansion media represents a clear limitation for the introduction of an ATMP at the clinical level. Currently, cell expansion is carried out in culture media supplemented with FBS. Serum use must be of a clinical grade (free of animal pathogens). Together with the growing demand for MSCs, this feature has led to a series of technical and ethical conditions for production (use of a large number of bovine fetuses) and geographic regions (zones free of prion diseases) with an associated impact on price (118–124). The substitution of FBS with human serum and platelet lysate also represents a technical limitation that is mainly related to the supply of human material and the absence of uniformity of the lots. Altogether, these considerations have enforced the development of robust processes of MSC production in chemically defined culture media free of animal and human components. These media are supplemented with recombinant proteins (albumin, insulin, TFGβ and bFGF), iron, selenium, and an antioxidant system (2-mercaptoethanol) (119, 122). The use of serum-free and xeno-free media minimizes the possible risks of contamination and adverse effects with respect to clinical application. Although several serum-free media have been described in the literature, they are chemically defined and contain known molecular components and are commercially available for cell manufacturing. We propose using our patented xeno-free and human component-free medium (WO2017021535) to expand modified MSCs.

## Cost of the Process

After the introduction of CAR-T cell therapies with an actual cost of ~300,000 to 400,000 € (125) or the prices charged by PROCHYMAL (an allogeneic bone marrow-derived allogeneic MSC treatment for graft vs. host disease) or Provenge (an autologous cell therapy of dendritic cells from metastatic forms of prostate cancer), with prices between $100,000 and $200,000 US, it seems absolutely necessary to analyze the cost-effectiveness of a potential treatment to facilitate the universal coverage of healthcare. A recent survey from the International Society for Stem Cell Therapy estimates costs for a dose between 10,000 and 25,000 € (126), with additional costs from hospitalization and the endovascular department, among others, resulting in a total cost of 30,000 to 40,000 € for a single dose. This cost may be assumed for rare diseases with a low prevalence, but it seems quite difficult to extend this treatment to a highly prevalent medical condition. The only way to reduce the cost is the mass production of allogeneic doses and facilitation of administration. Intramuscular administration of allogeneic MSCs will reduce the total cost and may be as effective as the intraarterial route. Given the reported adverse events, such as microthrombosis (19), clot formation (95), or IBMIR (82, 86, 96), the high cost of the treatment of complications and our preliminary data suggesting that allogeneic MSCs administered intramuscularly may be as safe and effective as intraarterial autologous MSCs.

We have now treated a huge number of patients with cell therapies, and the insights that we are gaining concerning the optimization of the next-generation of cell-based therapies should not be underestimated. We propose a combination among the following factors:

- A new source of healthy allogeneic donors (NOMA Project)
- A cost-effective mass production under GMP conditions (to be developed)
- A safe, friendly and less costly procedure for administration, for example, via the intramuscular route (NOMA Project)
- A new xeno-free culture medium (to be developed)

## Concluding Remarks

Considering all the previously findings and despite promising results, the ATMP-based therapy applied to CLI has led to important questions regarding safety and efficacy that can be transferred to its application for other pathologies. Although MSCs display a series of properties (factor release, immunomodulation, inflammatory capacity, among others), we presently do not know how many clinical trials are necessary before a specific, safe and effective cell-based therapy can be successfully achieved to offer patients with no other therapeutic alternatives. Improvements in personalized cell production in a cost-effective, safe and effective manner, a correct diagnosis, clinical prognosis and well-defined patient profile, and the correct route of administration will undoubtedly improve this advanced cellular medicine therapy.

## Author Contributions

BS conceived the concept of the paper. BS-J, NE, VC-G, VJ, MG-A, DG-O, AH, and BS wrote the first draft that was circulated and all the authors contributed with different sections. All the authors (BS-J, NE, VC-G, YA, LL, JT, FB, VJ, AD, RR-S, EA, LG, FP, FS-G, FL, MM, LD, GC, JM, RS, MG-A, DG-O, FM, AH, and BS) contributed to the acquisition, analysis, interpretation of data for the work, revising it critically for important intellectual content, final approval of the version to be published, and agree to be accountable for all aspects of the work in ensuring that questions related to the accuracy or integrity of any part of the work are appropriately investigated and resolved. AH edited and submitted the final version of the manuscript.

## Conflict of Interest

DG-O is a member of the Advisory Board of TiGenix SAU and received fees from Takeda. Authors filed several patents (US20160161504, WO2017032612, and WO2017021535) all related with the topic of this manuscript. The remaining authors declare that the research was conducted in the absence of any commercial or financial relationships that could be construed as a potential conflict of interest.
