# Corrigendum: Cost-Effective, Safe, and Personalized Cell Therapy for Critical Limb Ischemia in Type 2 Diabetes Mellitus

**DOI:** 10.3389/fimmu.2020.02029

**Published:** 2020-09-02

**Authors:** Bárbara Soria-Juan, Natalia Escacena, Vivian Capilla-González, Yolanda Aguilera, Lucía Llanos, Juan R. Tejedo, Francisco J. Bedoya, Verónica Juan, Antonio De la Cuesta, Rafael Ruiz-Salmerón, Enrique Andreu, Lukas Grochowicz, Felipe Prósper, Fermín Sánchez-Guijo, Francisco S. Lozano, Manuel Miralles, Lourdes Del Río-Solá, Gregorio Castellanos, José M. Moraleda, Robert Sackstein, Mariano García-Arranz, Damián García-Olmo, Franz Martín, Abdelkrim Hmadcha, Bernat Soria, the Collaborative Working Group “Noma Project Team”

**Affiliations:** ^1^Fundación Jiménez Díaz Health Research Institute, Madrid, Spain; ^2^Department of Regeneration and Cell Therapy, Andalusian Center for Molecular Biology and Regenerative Medicine (CABIMER), University of Pablo de Olavide-University of Seville-CSIC, Seville, Spain; ^3^Spanish Biomedical Research Centre in Diabetes and Associated Metabolic Disorders (CIBERDEM), Madrid, Spain; ^4^Andalusian eHealth Library, Sevilla, Spain; ^5^Unidad de Isquemia Crónica de Miembros Inferiores, Hospital Victoria Eugenia de la Cruz Roja, Sevilla, Spain; ^6^Servicio de Cardiología, Hospital Universitario Virgen Macarena, Sevilla, Spain; ^7^Clínica Universidad de Navarra, Pamplona, Spain; ^8^IBSAL-Hospital Universitario Salamanca, Salamanca, Spain; ^9^Department of Surgery, University of Valencia, Valencia, Spain; ^10^Cirugía Vascular, Hospital Universitario de Valladolid, Valladolid, Spain; ^11^Servicio Hematología y Hemoterapia, Hospital Clínico Universitario Virgen de la Arrixaca, Murcia, Spain; ^12^Herbert Wertheim College of Medicine, Florida International University, Miami, FL, United States; ^13^ISABIAL and Institute of Bioengineering, University Miguel Hernández de Elche, Alicante, Spain

**Keywords:** cellular medicaments, cell-based therapy, clinical trials, diabetes, critical limb ischemia, cost-effective

In the original article, there were multiple errors. The corresponding author emails karim.hmadcha@cabimer.es and bernat.soria@cabimer.es are no longer operational and should be replaced as follows:

Abdelkrim Hmadcha

khmadcha@gmail.com; khmadcha@upo.es

Bernat Soria

bernat.soria@umh.es; bsoresc@acu.upo.es

We further neglected to indicate the funder “Juvenile Diabetes Research Foundation, JDRF 2-SRA-2019-837-S-B I” to Bernat Soria.

In the published article, as well as having **Affiliations 2** and **3**, Bernat Soria should also have an additional affiliation “ISABIAL and Institute of Bioengineering, University Miguel Hernández de Elche, Alicante, Spain.”

Corrections have been made to the **References**. Reference 67 was incorrectly included as “Riera MLS A. A., Stefanov K. S., Tong H., Riera C. L., García-Olmo D., García-Arranz M. Phase Ib Open Clinical Trial to Assess the Safety of Autologous Mesenchymal Stem Cells for the Treatment of Nonrevascularizable Critical Lower Limb Ischemia. *J Stem Cell Res Ther*. (2017) 7:391.”. Instead, it should be “Riera ML, Salazar AA, Stefanov KS, Tong H, Riera CL, García-Olmo D, et al. Phase Ib open clinical trial to assess the safety of autologous mesenchymal stem cells for the treatment of nonrevascularizable critical lower limb ischemia. *J Stem Cell Res Ther*. (2017) 7:391. doi: 10.4172/2157-7633.1000391.”

Reference 86 was also included as “Escacena N. Cellular medication as a therapeutic alternative in chronic critical limb ischemia in diabetic patients without the possibility of revascularization. Dissertation Thesis. Sevilla Spain: University of Sevilla. (2016)”. This reference should be included as number 107 “Escacena N. *Cellular medication as a therapeutic alternative in chronic critical limb ischemia in diabetic patients without the possibility of revascularization* (Dissertation Thesis). University of Sevilla, Seville, Spain (2016).”

In the original article, there were multiple errors in the text. Removal of various sections is required due to legal issues. Figure 2, Figure 5, and Table 4 require removal.

**Paragraph 2** in the section “**The Use of MSCs”** on pages 8 and 9 requires removal.

A correction has been made to the section “**COST OF THE PROCESS”** on pages 13 and 14. The section has been revised as follows:

“After the introduction of CAR-T cell therapies with an actual cost of ~300,000 to 400,000 € (125) or the prices charged by PROCHYMAL (an allogeneic bone marrow-derived allogeneic MSC treatment for graft vs. host disease) or Provenge (an autologous cell therapy of dendritic cells from metastatic forms of prostate cancer), with prices between $100,000 and $200,000 US, it seems absolutely necessary to analyze the cost-effectiveness of a potential treatment to facilitate the universal coverage of healthcare. A recent survey from the International Society for Stem Cell Therapy estimates costs for a dose between 10,000 and 25,000 € (126), with additional costs from hospitalization and the endovascular department, among others, resulting in a total cost of 30,000 to 40,000 € for a single dose. This cost may be assumed for rare diseases with a low prevalence, but it seems quite difficult to extend this treatment to a highly prevalent medical condition. The only way to reduce the cost is the mass production of allogeneic doses and facilitation of administration. Intramuscular administration of allogeneic MSCs will reduce the total cost and may be as effective as the intraarterial route. Given the reported adverse events, such as microthrombosis (19), clot formation (95), or IBMIR [82, (86, 96)], the high cost of the treatment of complications and our preliminary data suggesting that allogeneic MSCs administered intramuscularly may be as safe and effective as intraarterial autologous MSCs.”

The section “INSTITUTIONAL REVIEW BOARD AND REGULATORY COMPLIANCE” and its sub-sections “Clinical Trials” and “Regulatory and ATMP Manufacturing” on page 15 require removal.

As a result, two figures require renumbering. A correction has been made to Figure 3 and Figure 4 on pages 11 and 12. The figures should now state the following:

“Figure 2. Instant blood-mediated inflammatory reaction” and “Figure 3. Implications of MSCs for thrombosis risk during cell therapy”.

The authors apologize for these errors and state that this does not change the scientific conclusions of the article in any way. The original article has been updated.

## References

[86] Moll G Hult A von Bahr L Alm JJ Heldring N Hamad OA. Do ABO blood group antigens hamper the therapeutic efficacy of mesenchymal stromal cells? PLoS ONE. (2014) 9:e85040. 10.1371/journal.pone.0085040 24454787 PMC3890285

[95] George MJ Prabhakara K Toledano-Furman NE Wang YW Gill BS Wade CE. Clinical cellular therapeutics accelerate clot formation. Stem Cells Transl Med. (2018) 7:731–9. 10.1002/sctm.18-0015 30070065 PMC6186273

[96] Moll G Ankrum JA Kamhieh-Milz J Bieback K Ringden O Volk HD. Intravascular mesenchymal stromal/stem cell therapy product diversification: time for new clinical guidelines. Trends Mol Med. (2019) 25:149–63. 10.1016/j.molmed.2018.12.006 30711482

